# Chronic neutrophilic leukemia: new science and new diagnostic criteria

**DOI:** 10.1038/s41408-018-0049-8

**Published:** 2018-02-13

**Authors:** Natasha Szuber, Ayalew Tefferi

**Affiliations:** Department of Internal Medicine, Division of Hematology, Mayo Clinic, Rochester, Minnesota USA

## Abstract

Chronic neutrophilic leukemia (CNL) is a distinct myeloproliferative neoplasm defined by persistent, predominantly mature neutrophil proliferation, marrow granulocyte hyperplasia, and frequent splenomegaly. The seminal discovery of oncogenic driver mutations in *CSF3R* in the majority of patients with CNL in 2013 generated a new scientific framework for this disease as it deepened our understanding of its molecular pathogenesis, provided a biomarker for diagnosis, and rationalized management using novel targeted therapies. Consequently, in 2016, the World Health Organization (WHO) revised the diagnostic criteria for CNL to reflect such changes in its genomic landscape, now including the presence of disease-defining activating *CSF3R* mutations as a key diagnostic component of CNL. In this communication, we provide a background on the history of CNL, its clinical and hemopathologic features, and its molecular anatomy, including relevant additional genetic lesions and their significance. We also outline the recently updated WHO diagnostic criteria for CNL. Further, the natural history of the disease is reviewed as well as potential prognostic variables. Finally, we summarize and discuss current treatment options as well as prospective novel therapeutic targets in hopes that they will yield meaningful improvements in patient management and outcomes.

## Introduction: “An uncommon myeloproliferative disorder”

Chronic neutrophilic leukemia (CNL) is a rare *BCR*-*ABL* negative myeloproliferative neoplasm (MPN) characterized by sustained, predominantly mature neutrophil proliferation, bone marrow granulocytic hyperplasia, and hepatosplenomegaly. It was first described by Tuohy in 1920 in a report entitled “A case of splenomegaly with polymorphonuclear neutrophil hyperleukocytosis”^1^, detailing the case of a 58-year-old woman presenting with splenomegaly and an associated “overwhelming preponderance of mature polymorphonuclear neutrophils”. Subsequently, in 1932, Emil-Weil and See^2^ reported two more possible cases from European literature. Over 30 years later, Jackson and Clark reported on a rare case of MPN, coining the term neutrophilic leukemia^3^, but the inaugural use of the term “chronic neutrophilic leukemia” was finally attributed to Tanzer et al. in the Lancet in 1964^4^ and was re-iterated shortly thereafter by Rubin in the Annals of Internal Medicine in 1966^5^. Importantly, and perhaps disappointingly, whether these accounts represent actual cases of CNL remains uncertain as literature were scant and diagnostic criteria virtually non-existent.

As early findings consisted chiefly of isolated case reports or small case series, CNL was born of an era of being defined primarily by what it *was not*: by excluding basophilia, monocytosis, or a *BCR-ABL* fusion, it could be distinguished from chronic myeloid leukemia (CML), atypical chronic myeloid leukemia (aCML), and chronic myelomonocytic leukemia (CMML). Similarly, by eliminating potentially confounding underlying clinical conditions such as infections or malignancy it could, sometimes with difficulty, be differentiated from a leukemoid reaction.

Through the years, CNL has regrettably suffered an identity crisis of sorts, even embodying a challenge of nomenclature. In a comprehensive review from 2002 published in the British Journal of Haematology^6^, Reilly distinguishes “true CNL” from potential mimickers such as “neutrophilic-chronic myeloid leukemia”, or N-CML, “plasma cell dyscrasia-associated neutrophilia”, and “myelodysplastic-chronic neutrophilic leukemia,” emphasizing the obstacle of identifying cases of diagnostically pure CNL from a virtual diagnostic wastebasket. Even the terms used to describe the disease, themselves, have evolved from “neutrophilic leukemia” to “true chronic neutrophilic leukemia”^6^ to simply “chronic neutrophilic leukemia,” the current accepted World Health Organization (WHO)-defined diagnostic entity. In his statement: “the literature… is frequently confusing and often incomplete, with the result that CNL’s natural history and prognosis remain obscure,” Reilly thoughtfully articulated the magnitude of the gap in the state of scientific knowledge of CNL in 2002 and emphasized the need for a more stringent and definitive diagnostic framework^6^.

Accordingly, only a fraction of historically reported cases of “CNL” are actually consistent with present-day diagnostic criteria^6^, and in retrospect, many were probably erroneously labeled CNL when in fact representing cases of reactive neutrophilia or other myeloid malignancies.

The first proposed basic, albeit operational, diagnostic criteria for CNL date back to 1979 in a report by You and Weisbrot^7^. The elements considered central to CNL diagnosis were severe sustained mature neutrophilic leukocytosis, hepatosplenomegaly, absence of leukemoid reaction, and elevated values for leukocyte alkaline phosphatase (LAP), serum vitamin B12, and uric acid. However, it was not until 2001 that the WHO Classification of Neoplastic Diseases formally acknowledged CNL as a distinct myeloproliferative malignancy and included it as an entity in their Proposed WHO Classification of Myeloid Neoplasms^8,9^.

Increasing recognition of this rare MPN along with improvements in the diagnostic approach has prompted a progressive rise in the number of recorded cases of CNL with time. In 1979, only 13 cases had been reported^7^, and by 1996, there were still fewer than 100 altogether^10^. By 2002, 129 cases of CNL were described in the literature^11^ and by 2005, there were 150^12^. At the present time, over 200 cases of the disease are recorded, although the true occurrence of CNL is likely much lower when strict WHO diagnostic criteria are applied. This has recently been detailed in a review by Bain and Ahmad^13^ counting a total of 52 published cases of “CNL” not meeting WHO diagnostic criteria for the disease.

## New science

The discovery of colony-stimulating factor 3 receptor (*CSF3R*) mutations in the majority of CNL patients^14^ revolutionized our understanding of the pathogenesis of this disease, provided a biomarker for CNL diagnosis, and paved the way for therapeutic targeting with small-molecule tyrosine kinase inhibitors (TKI). This landmark finding appropriately marked the beginning of a new era for CNL as it shifted our focus toward genomics at the heart of this once biologically obscure disease. With the subsequent identification of additional genetic lesions involved in CNL oncogenesis, some of which are potentially druggable targets, this molecular revolution in CNL conclusively marked the birth of new science for this once poorly defined disease entity. In light of these new findings, the aim of this article was to critically review available literature, highlight recent advances in molecular diagnostics, present updated diagnostic criteria, and outline current as well as novel and developing therapeutic approaches.

## Epidemiology

The increasing number of CNL cases being reported has enabled a more accurate assessment of its epidemiology; however recognizing that many of these represent “mimickers” and considering the still relatively scarce literature, the epidemiology of the disease remains imperfectly defined. According to the Surveillance, Epidemiology, and End Results (SEER) Program of the National Cancer Institute, CNL remains a rare disease and its true incidence is unknown^15^. It typically presents in elderly patients, with 88% of CNL patients being over age 50 in one report^11^. The median age at diagnosis was 66.5 years (range: 15–86) in one of the largest series of 40 WHO-defined CNL cases^16^. Curiously, a recent publication by Ouyang et al. found a much younger median age of 39 years (range 27–92 years) in their 10-patient CNL cohort^17^ which may be biased by a small sample size. Sex distribution was thought to be equal although more recent data shows a slight male preponderance of 56–57% as revealed by a series of 40 CNL patients and 14 molecularly defined CNL cases, respectively^16,18^. Interestingly, there is also some evidence for a preponderance of males specifically in the *CSF3R*-mutated cohort of CNL patients^17,19^.

## Clinical features

CNL is a clinically heterogeneous disease which may present as incidental neutrophilic leukocytosis on a routine blood count or alternatively with a spectrum of constitutional symptoms including fatigue, weight loss, night sweats, bone pain, easy bruising, pruritus, or gout^10^. The majority of patients are asymptomatic at diagnosis. Of presenting clinical manifestations, fatigue appears to be the most common. In a series of 14 molecularly defined CNL patients, 71% had antecedent chronic leukocytosis lasting a median of 12.5 months (range 5–84 months)^18^. Clinical examination may reveal splenomegaly (present in 36% in one series^18^) involving primarily red pulp cords and sinuses, and/or hepatomegaly, but as in CML, lymph node involvement remains relatively uncommon^20^.

There is substantial evidence for an association of CNL with a hemorrhagic diathesis. Reports as early as 1979 describe an “unexplained hemorrhagic tendency” in a portion of CNL patients^7^ which has been reiterated in later reports^11,21–23^, including a considerable number of cases of cerebral hemorrhage. In a report by Bohm, of 14 new cases of CNL reviewed of which 10 had died (mean survival time 14.7 months), the most frequent cause of death was in fact cerebral hemorrhage, seen in six cases^11^. Mechanistically, this bleeding diathesis has been attributed to thrombocytopenia, platelet dysfunction, and/or potentially to vascular wall infiltration by neoplastic cells^22,24^. It has been suggested that patients displaying bleeding complications out of proportion to their platelet count be evaluated for acquired von Willebrand’s disease and other acquired coagulation and platelet function disorders.

## Laboratory findings

The hallmark of CNL is sustained, chronic, mature neutrophilia. In a series of WHO-defined CNL, the median leukocyte count was 39 × 10^9^/L (range: 11–126) with predominant neutrophilia^12^. Importantly, CNL is characterized by the presence of more mature granulopoietic forms compared with CML, with granulocytes nearly exclusively (≥80%) at the segmented and band stage of development and minimal to no circulating blasts. The notable absence of monocytosis, basophilia, and eosinophilia is another feature of CNL. The majority of CNL patients present with mild anemia (median hemoglobin ~11 g/dL)^12,16^ and/or thrombocytopenia. More commonly, thrombocytopenia is reported with disease progression along with increasing splenomegaly, often heralding blast crisis^12^. Lactate dehydrogenase (LDH) levels are typically elevated, as is the LAP score; this latter observation remains non-specific but contrasts with the low LAP score noted in CML. Vitamin B12 levels are also often elevated due to transcobalamin release from granulocytes and their precursors^10^. Interestingly, low serum granulocyte-colony stimulating factor (G-CSF) levels have been documented in CNL patients by several groups^25–27^ though this test is not readily available.

## Bone marrow morphology

Bone marrow is uniformly and intensely hypercellular in CNL due to expanded neutrophilic granulopoiesis with an increased myeloid to erythroid ratio which may be in excess of 20:1^28^. However, the left shift is distinctly more subtle and mature neutrophils more prominent than in CML with the majority of granulocyte precursors being at the metamyelocyte to segmented stages of maturation. Fewer than 5% myeloblasts are present and there is an absence of Auer rods as well as no dysplastic features^16^. Reticulin staining, though characteristically normal, may show minimal fibrosis but should not exceed a grade of 1+^12^. Erythroid maturation is normoblastic and megakaryocytes, while morphologically normal, may be normal or slightly increased in number^16^.

## Neutrophil morphology

Neutrophil toxic granulations and Dohle bodies are a common finding in CNL and may be suggestive of an activated neutrophil state. It is of note that these same findings are more frequently observed in neutrophilic leukemoid reactions but remain non-specific^13^. While some reports have shown normal neutrophil chemotaxis in CNL^29^, others demonstrate increased phagocytosis and superoxide production suggesting increased neutrophil function^25^. Paradoxically other studies have shown attenuated intracellular bactericidal activity in CNL^25,30^. Whether this biological phenomenon translates clinically to an increased propensity for infection in CNL patients is not clear^31,32^.

## Genetic predisposition

To date there is a single report in the literature of a familial case of suspected CNL involving a father and son who both developed the disease^33^. The father had previously been exposed to radiation following the atomic bomb attack on Hiroshima however, and the oncogenetic effect of this remains a potentially confounding pathogenic factor.

## Differential diagnosis

Neutrophilic leukocytosis may underlie a wide variety of diseases, both benign and malignant. Furthermore, the clinical and laboratory features of CNL may overlap with these disorders. Given that an accurate diagnosis of CNL requires exclusion of a number of mimickers, it is critical to consider potentially confounding diagnoses such as reactive neutrophilia/leukemoid reaction, CML, CML-N, MPN/MDS (myeloproliferative neoplasms/myelodysplastic syndromes) overlap disorders such as aCML and CMML, as well as other myeloid neoplasms.

Distinguishing CNL from a leukemoid reaction can be challenging given that both may present with significant neutrophilia, bone marrow hypercellularity, normal cytogenetics, and absence of *BCR-ABL* fusion gene. WBC count may be more modestly elevated in leukemoid reaction, though there have been some reports of WBC counts up to 100 × 10^9^/L in this context^34^. Meticulous history-taking along with a thorough clinical examination to exclude alternate diagnoses, including occult malignancy or infection, and a period of observation is often recommended before formally determining the diagnosis. The demonstration of clonality including identification of a *CSF3R* mutation or other molecular or cytogenetic abnormality is clearly valuable in aligning the diagnosis in favor of CNL.

CML is invariably associated with a *BCR-ABL* fusion gene and displays a disproportionally higher percentage of myelocytes. CML also commonly presents with basophilia, thrombocytosis, or eosinophilia. While the absence of the Philadelphia chromosome is implicit to CNL diagnosis, a rare form of CML termed neutrophilic-CML, or CML-N, has been described which shares morphological features of CNL, specifically a prominent neutrophilic proliferation^35^. CML-N, however, is characterized by an uncommon *BCR-ABL* translocation which results in the transcription of an e19/a2 type *BCR-ABL* messenger RNA yielding a 230-kD *BCR-ABL* protein (p230)^12,36^. The clinical correlate is a lower total WBC count, less severe anemia, less prominent splenomegaly, and blastic transformation occurring much later in CML-N patients^35^. The attenuated phenotype and indolent course of CML-N, specifically in patients without additional cytogenetic abnormalities, is now postulated to be due to low p230 mRNA and protein levels^37^. The diagnosis of CNL also requires that molecular testing be negative for defining markers of alternate neoplasms including not only the *BCR-ABL1* fusion gene but also rearrangements in *PDGFRA/B* or *FGFR1*, characteristic of eosinophilic leukemia.

Atypical CML represents an uncommon and heterogeneous disease with overlapping features of both myeloproliferative and myelodysplastic neoplasms. It is characterized by leukocytosis (neutrophils and precursors comprising ≥10% of leukocytes) and prominent granulocytic dysplasia, useful in distinguishing it from CNL. Atypical CML also does not meet criteria for *BCR-ABL*-positive CML, demonstrates absence of *PDGFRA/B* and *FGFR1*, and presents with minimal basophilia and monocytosis.

CMML is another MPN/MDS overlap disorder whose diagnosis requires persistent (≥3 months) peripheral monocytosis >1 × 10^9^/L, absence of *BCR-ABL* fusion or *PDGFRA/B* rearrangements, fewer than 20% blasts or promonocytes in blood and bone marrow, and evidence of dysplasia or clonal abnormality (or persistent monocytosis lasting ≥3 months with exclusion of all other causes). The chronic monocytosis and presence of dysplasia in CMML are central in distinguishing it from CNL.

Other myeloid malignancies must also be excluded, including polycythemia vera (PV), essential thrombocythemia (ET), and primary myelofibrosis (PMF) which each display characteristic genetic and morphological features and whose diagnostic criteria are well outlined in the revised 2016 WHO classification of myeloid neoplasms^38^.

Very rarely, paraneoplastic leukocytosis may result from ectopic production of G-CSF by certain solid tumors, mimicking the neutrophilic leukocytosis of CNL. Such cases are described primarily in association with urological malignancies such as renal cell carcinoma^39^ but have also been reported in other tumor types including lung^40^, mesothelium^41–43^, thyroid^44^, sarcoma^45^, adrenal carcinoma^46^, stomach^47^, gallbladder^48^, and malignant melanoma^49^. The prognostic relevance of tumor-associated G-CSF production is uncertain, though some authors have suggested it may portend a poorer prognosis owing to G-CSF’s role in autocrine growth stimulation, resulting in more aggressive tumor growth^46^.

## Disease associations

Several reports have found associations between CNL and other clonal myeloid and lymphoid disorders with the two most commonly described being polycythemia vera and plasma cell disorders. Though the presence of a concomitant clonal hematologic disease may confound the underlying diagnosis of CNL, there is some literature to convincingly support some of these associations.

### Polycythemia vera

There have been multiple reports describing an association between CNL and PV. These have ranged from accounts of concomitant CNL and PV to cases of PV terminating in a CNL phenotype^26,50–56^. However, not only were patients exposed to cytoreductive therapy in virtually all cases published, raising the question of whether these cytotoxic agents may have contributed to CNL pathogenesis, but the number of reports is too limited to draw definitive conclusions. Furthermore, in some such cases, patients harbored comorbidities potentially associated with reactive neutrophilia, including concomitant malignancies, challenging the very diagnosis of CNL. Thus, although not all cases of CNL–PV represent true associations, those that do may lend support to the clonal origin of CNL as well as its classification among the MPN.

### Plasma cell disorders

A disproportionately high number of cases of CNL associated with plasma cell neoplasms have been reported considering the rarity of this disease^57–65^, even prompting the distinction of a “monoclonal gammopathy-associated CNL” or MG-CNL entity. Plasma cell dyscrasias have been shown to occur in up to 32% of CNL cases^66^. In a recent review on this subject, after excluding suspected cases of CNL not meeting WHO criteria, ultimately, 49 patients with “CNL” or neutrophilic leukemoid reaction associated with multiple myeloma or MGUS were found in the literature^13^. The associated plasma cell dyscrasia was multiple myeloma in the majority of cases, followed by MGUS and rarely plasmocytoma^13^. The majority of patients expressed lambda light chain with the remainder, kappa, and rarely both^13^, supporting previous reports of predominantly lambda light chain restriction in CNL-associated plasma cell dyscrasias^63^.

The fundamental question plaguing this relationship has remained whether the CNL and plasma cell disorders are clonally related or whether the neutrophilia is secondary to the underlying plasma cell neoplasm. Erber and Reilly raised this question in their aptly titled commentary “Chronic neutrophilic leukemia with plasma cell dyscrasia: friends or relatives?”^67^. Overall, three main hypotheses have emerged: firstly, the possibility of a common progenitor cell differentiating along both myeloid and lymphoid lines resulting in a biphenotypic neoplasm; secondly, the co-existence of two independent clonal disorders; and thirdly, a purely reactive neutrophilia associated with the plasma cell dyscrasia. The aforementioned review by Bain et al. asserts that leukocytosis in suspected concurrent neoplasms is attributable, in the majority of cases, to cytokine-driven neutrophilia accompanying the primary plasma cell neoplasm^13^ and there is substantial evidence corroborating this, including demonstration of high G-CSF levels and neutrophil counts in a patient with myeloma whose levels declined upon treatment with steroids^68^. Moreover, several other studies have confirmed G-CSF synthesis by neoplastic plasma cells^69–74^. Furthermore, there are reports supporting the polyclonal nature of the plasma cell dyscrasia-associated neutrophilia, cases of spontaneous remission of the presumed CNL, and even improvements in neutrophil count and hepatosplenomegaly following treatment of the underlying dysproteinemia^62,63,72,75^. Another important distinction is a prognostic one as MGUS-associated CNL patients appeared to have superior survival when compared to strictly WHO-defined CNL^75,76^ suggesting different etiopathogeneses. The fact that Pardanani et al. did not find *CSF3R* mutations in five cases of plasma cell neoplasm-associated “CNL” also suggests that both entities are distinct^76^. In addition, the fact that MGUS is common within the age range in which CNL characteristically occurs further supports a merely incidental association. As a whole, this data argue in favor of polyclonal neutrophilic proliferation *secondary to* antecedent or underlying plasma cell disorder. Consequently, any suspected association should be confirmed by demonstrating neutrophil clonality.

Challenging this precept, Nedeljkovic et al. provided evidence that the neutrophilia in one case of paraproteinemia in a patient with CNL was clonal and not reactive^77^. This individual harbored a *JAK2*V617F mutation, establishing the clonal nature of the myeloid proliferation. Another similar case of *JAK2*V617F-positive CNL with MGUS was subsequently reported^76^. There is also an account of emergence of *CSF3R* mutations during follow-up of a patient with MGUS, representing the first case of a plasma cell neoplasm associated with CNL as defined by this highly sensitive and specific clonal marker^78^. More recently, *SETBP1* mutations (D868N and G870S) were identified in two of five cases of neutrophilia with a concomitant plasma cell neoplasm, also suggesting a separate clonal myeloid entity in these individuals^79^. These cases thus support clonal neutrophilia concurrent with plasma cell dyscrasia in at least a portion of described reports based on the critical finding of a clonal myeloid marker.

Altogether, though the nature of the putative CNL-dysproteinemia relationship remains controversial, it is likely that both clonal and non-clonal neutrophilia are possible in association with plasma cell neoplasms. The WHO once recommended that all suspected cases of CNL be screened for plasma cell disorders^28^ and that if present, neutrophil clonality be demonstrated via cytogenetic or molecular techniques in order for the hypothesis of CNL to be viable; however, with the current availability of the sensitive and specific CSF3R marker, as will be discussed, this is no longer required.

### Other associations

Rare reports have shown CNL evolving from MDS^80^ as well as an association with chronic lymphocytic leukemia^81^ but these accounts remain anecdotal. More recently, Wang et al. reported a case of biopsy-proven nephrotic syndrome (kidney biopsy positive for *JAK2*V617F) related to *JAK2*-positive CNL successfully treated with Hydroxyurea followed by Hydroxyurea combined with Interferon^82^.

## Molecular cytogenetics and clonality

Evidence for clonality in CNL was initially supported by the finding of a monoclonal methylation pattern of the X-linked hypoxanthine phosphoribosyl transferase (HPRT) gene in a patient with CNL^83^. Other groups subsequently demonstrated the clonal nature of the leukemic neutrophils in CNL using HUMARA clonality studies^84^ and fluorescent in situ hybridization (FISH)^85^. Although the majority of patients with CNL display normal cytogenetics at diagnosis^12,28^ the demonstration of non-specific cytogenetic abnormalities in a proportion of CNL patients served as later proof of clonality.

A report by Didonato et al. was the first to demonstrate cytogenetic aberrations in the form of trisomy 9 and a partial deletion of the long arms of chromosome 20 in a patient with suspected CNL^86^. Ensuing reports confirmed the occurrence of cytogenetic abnormalities in a minority of CNL patients at diagnosis and/or during clonal evolution^6,12,87^. In Reilly’s 2002 review, 37% of CNL cases presented abnormal cytogenetics consisting primarily of trisomy 8, trisomy 21, deletion 11q, and deletion 20q^6^. Subsequently one of the largest CNL case series (total of 40 CNL patients) identified cytogenetic abnormalities in 13 of 40 patients (32.5%)^12^. These aberrations were detected at baseline in 20% of patients and during clonal evolution in the remaining 12.5% and included deletion 20q, trisomy 21, deletion 11q, and deletion 12p^12^. Though the most common chromosomal lesions in CNL consist of trisomy 8 and deletion 20q, observed either at diagnosis or at the time of clonal evolution^18^, multiple additional abnormalities have been described including tetraploidy 21, trisomies 7, 8, and 9, translocation 1;20, deletion Y, deletion 6, add 5p, deletion 15, and monosomy 2^88^, and are considered non-specific, yet non-random findings in myeloid neoplasms^89^.

In rare cases, detection of a well-known MPN molecular marker such as *JAK2*V617F may also serve to establish clonality^12,90^.

## Molecular pathogenesis and diagnosis

### *CSF3R* mutations

G-CSF is a cytokine growth factor that regulates basal as well as stimulated “emergency” granulopoiesis and promotes granulocyte differentiation^91^. G-CSF is secreted principally by macrophages, fibroblasts, and endothelial cells. The pivotal role of this mitogenic cytokine in granulopoiesis was underscored as CSF3 or CSF3 receptor (CSF3R)-deficient mice were found to be severely neutropenic^92,93^. The G-CSF receptor, CSF3R, is a single chain cell-surface receptor belonging to the cytokine receptor type I superfamily whose gene maps on chromosome 1p34.3 and consists of 17 exons^91^. Its cytoplasmic domain contains distinct functional regions with the membrane-proximal region being functionally ascribed to a mitogenic signaling function while the carboxy terminal is involved in maturation signaling and regulation/suppression of proliferation^94^.

Upon binding to its receptor, G-CSF exerts its effects via classical downstream pathways Janus kinase (JAK)-signal transducer and activator of transcription (STAT), SRC family kinases (notably LYN)^95,96^, non-receptor tyrosine kinase SYK, Ras/Raf/MAP kinases, and PI3K/Akt pathways, inducing neutrophilic differentiation, proliferation, and survival as well as stimulating neutrophil function^97^. The discovery of the colony-stimulating factor 3 receptor (*CSF3R*) mutations in the majority of CNL patients represents a turning point in our understanding of the molecular pathogenesis of this disease^14^. Before this seminal finding, however, *CSF3R* mutations were already recognized in several forms of myeloid disorders including severe congenital neutropenia (SCN), hereditary chronic neutrophilia, and rarely in myeloid leukemias, as will briefly be discussed below.

## Severe congenital neutropenia

SCN is a clinically heterogeneous inherited condition characterized by early onset of severe bacterial infections. Acquired somatic truncating mutations in *CSF3R*, analogous to those found in CNL^14^, have been described in up to 30–40% of patients with SCN^98^ concurrently with inherited, pathogenically relevant mutations such as *ELANE* or *HAX1*^99^. Though these acquired, mostly non-sense *CSF3R* mutations were initially thought to contribute to SCN pathogenesis^100,101^, they were later demonstrated to molecularly annotate a subset of ~80% of SCN patients at high risk for leukemic conversion^98,101–105^. These truncating mutations, which lead to loss of carboxy-terminal regulating domain, are neither required nor sufficient for induction of leukemia in SCN, as supported by knock-in murine models^106^. It is currently postulated that through co-operation with other oncogenes^103,107^, they provide a clonal advantage of prolonged cell survival^108^ favoring the acquisition of additional oncogenic “hits” ultimately resulting in a leukemic phenotype^106^.

Interestingly, in 2012, Beekman et al. reported a novel acquired membrane proximal *CSF3R* autoactivating mutation, now known to be *CSF3R*T618I (also referred to as C*SF3R*T595I if the 23-amino-acid signal peptide is excluded from the numbering of amino acids), in a patient with SCN having developed AML 17 years after initiating G-CSF treatment (in this case co-occurring with a *CSF3R* truncating mutation)^103^. The same mutation was also reported in one of 199 other AML cases published by the Cancer Genome Atlas^103^, highlighting abnormal G-CSF signaling as a potential driver of leukemic transformation.

## Hereditary chronic neutrophilia

Another distinct *CSF3R* mutation has been involved in the pathogenesis of hereditary neutrophilia. The germline, autosomal dominant transmembrane domain *CSF3R*T617N mutation was described by Plo et al.^109^ and was found to induce dimerization of the CSF3R transmembrane domain, promoting constitutional activation of the receptor in a family with chronic neutrophilia (12 affected individuals out of 16, over 3 generations). The *CSF3R*T617N mutation induced receptor-independent granulocyte proliferation and downstream constitutive phosphorylation of JAK2, STAT3, AKT, and ERK which was sensitive to JAK2 inhibition^109^. In the affected family, neutrophilia was accompanied by splenomegaly and increased circulating CD34-positive myeloid progenitors, with only the proband progressing to myeloid malignancy (MDS)^109^. This report underscores the importance of seeking out a family history of neutrophilia in suspected cases of CNL in order to exclude a hereditary basis.

## Leukemia

Incidentally, the same membrane-proximal *CSF3R*T617N mutation found in hereditary neutrophilia was first described as a possible polymorphism in AML in 1996^110^ and later confirmed as a leukemia-specific mutation in two of 555 AML patients;^111^ it is also rarely found in CNL^112^.

Zhang et al. recently identified a novel activating mutation (W341C) in the extracellular domain of CSF3R in a leukemic patient, coexpressed with a truncation mutation (W791X)^113^. In vitro studies demonstrated W341C-induced constitutive activation of JAK-STAT and ERK, G-CSF independent cell growth, and transformation of BaF3 cells which translated clinically to progressive leukocytosis and thrombocytopenia. The oncogenic mechanism of W341C was attributed to unpaired cysteine-mediated disulfide bond formation leading to CSF3R dimer stabilization^113^.

## Pediatric AML

In recent years, *CSF3R* mutations have been identified at a rate of 2.4% in pediatric AML patients and found to exhibit a strong association with *CEBPA* mutations^114^. In this setting, patients with transforming *CSF3R* mutations have displayed a trend toward low risk disease, which has been ascribed to their association with the more favorable *CEBPA* mutations.

## *CSF3R* mutations in CNL: “The *CSF3R* mutations represent a biologically unifying feature of CNL”^14^

In 2013, a landmark study by Maxson et al. was published identifying disease-defining oncogenic *CSF3R* mutations in the majority of patients with CNL^14^. This report heralded a new era in the history of CNL as it elucidated its molecular pathobiology, provided a biomarker for diagnosis, and rationalized the therapeutic use of JAK inhibitors. In this study, *CSF3R* mutations were found in 89% and 40% of CNL and aCML cases, respectively. Two mutational variants of CSF3R were identified: the more common membrane proximal mutations consisting primarily of T618I and T615A point mutations, and frameshift or non-sense mutations leading to premature truncation of the cytoplasmic tail of CSF3R (D771fs, S783fs, Y752X, and W791Z). The truncation mutations, which also occur in SCN, typically coexisted with membrane proximal or transmembrane CSF3R mutations as compound mutations^14,115^. Mechanistically, the membrane proximal mutations prevent *O*-glycosylation of CSF3R resulting in increased active dimeric configuration, ligand-independent receptor activation, and subsequent constitutive downstream signaling through JAK2^14,116^. Alternately, several mechanisms may explain how truncation mutations lead to uncontrolled cellular proliferation. Truncation of the receptor involves a loss of negative regulatory motifs including the dileucine sorting motif, which plays a role in receptor internalization^117^, and binding sites for the suppressor of cytokine signaling 3 (SOCS3) which normally targets the receptor for degradation^118^. Truncation mutations thus disrupt receptor trafficking and result in delayed receptor internalization and/or degradation^115,117^, increased cell surface expression of truncated CSF3R, sustained STAT5 activity, and enhanced cell proliferation^119^.

The capacity of the most common CSF3R mutation, *CSF3R*T618I, to drive leukemogenesis in CNL was established by Fleishman et al. using a murine bone marrow transplant model^120^. The *CSF3R*T618 mutation induced a lethal myeloproliferative disorder which recapitulated the clinical features of CNL, supporting the role of *CSF3R*T618I as an oncogenic driver mutation and providing “proof of concept” to Maxson et al.’s seminal report^120^. Moreover, the *CSF3R*T618I mutation was confirmed to act via the JAK-STAT signaling pathway as the associated pathogenic granulocytosis and splenomegaly were sensitive to treatment with JAK inhibitor Ruxolitinib^120^. An analogous study demonstrated the leukemogenic potential and likewise favorable response to Ruxolitinib of one of the alternate transmembrane domain mutations, *CSF3R*T640N, which behaves similarly to *CSF3R*T618I^112^.

The proportions of CNL/aCML patients harboring membrane-proximal only versus membrane-proximal concurrent with a truncating CSF3R mutation (compound mutant) are 75% and 25%, respectively^121^. In their consideration of the scarcity of truncation-only *CSF3R* mutations in CNL, Maxson and Tyner raised the possibility that, contrary to membrane proximal mutations, truncation variants alone may be insufficient to drive leukemogenesis in CNL^122^. Consistent with this, truncation mutations display slower cell transformation kinetics in cellular assays and require ligand for activation of downstream signaling^14^. Finally, studies using knock-in mice expressing truncated *CSF3R* confirmed that these mutations in isolation were insufficient to induce leukemic transformation^106^. The biological basis for this attenuated pathogenicity of truncation mutations has recently been reasoned to be due to inefficient MAP kinase signaling^123^, providing a mechanistic explanation as to why *CSF3R* truncation mutations are usually found concurrently with other mutations such as T618I or T615A in CNL^14,121^.

Importantly, in Maxson et al.’s landmark study, the two prototypical *CSF3R* mutations showed differential susceptibilities to different TKIs: membrane proximal mutations responded preferentially to the JAK inhibitor Ruxolitinib while truncation mutations displayed sensitivity to inhibition with SRC kinase inhibitor Dasatinib^14^. This key finding validated the concept of distinct downstream signaling dysregulation in CNL involving either JAK-STAT or SRC kinase pathways and provided a rationale for molecularly directed therapeutic targeting with TKI. This study also confirmed the low frequency of *CSF3R* mutations in AML (later reaffirmed by Beekman et al., finding a prevalence of *CSF3R* mutations of <0.5% in a series of 1446 consecutive cases of de novo AML^124^), and their absence in B-cell and T-cell ALL as well as in reactive neutrophilia^14,76^, suggesting that the *CSF3R* mutation represents the defining molecular lesion in CNL.

A follow-up study by Pardanani et al. took the approach of dividing patients into broad groups according to strictly WHO-defined disease subsets^76^. Mutations in *CSF3R* were analyzed in 35 suspected cases of CNL, 12 of which were subsequently confirmed to have WHO-defined CNL. Significantly, *CSF3R* mutations were detected in 100% of WHO-defined CNL cases and none of the cases of WHO-defined aCML, MG-associated CNL, PMF or CMML^76^. *CSF3R*T618I mutations were the most prevalent, detected in 10 of 12 CNL patients for a mutational frequency of 83%, while the remaining two patients harbored alternative *CSF3R* mutations M696T and I598I^76^. This study thus critically endorsed the *CSF3R*T618I mutation as a sensitive and specific molecular marker for WHO-defined CNL and the authors consequently recommended its incorporation into current diagnostic criteria^76^.

Subsequent studies have both corroborated the lower mutational frequency of *CSF3R* mutations in aCML, which overall ranges from 0–40%^14,19,125^, and confirmed the high prevalence of *CSF3R* mutations in WHO-defined CNL, identifying these mutations in 80–100% of patients as shown by Cui et al. and Ouyang et al.^17,126^. In this latter publication, seven of the eight patients carried the *CSF3R*T618I mutation (including two harboring compound mutations), while the remaining patient displayed a *CSF3R*P733T mutation. This study supported the occurrence of *CSF3R*T618I mutations exclusively in WHO-defined CNL and validated this mutation as a sensitive and specific diagnostic marker in CNL^17^. Interestingly, this study also found *CSF3R*-mutated patients to have higher hemoglobin levels compared to their wild-type counterparts, a phenotypic characterization that remains to be validated^17^. Though the majority of data supports CSF3R as a sensitive and specific marker of CNL, variable mutational frequencies have been reported. Meggendorfer et al. found that only six of 14 CNL patients (43%) harbored *CSF3R* mutations and additionally identified *CSF3R* mutations in two of 58 (3.4%) aCML patients and two of 146 (1.4%) CMML patients^19^. This discrepancy is likely due to small sample sizes, non-uniformity in adopting strict WHO-defined diagnostic criteria, and inconsistencies in morphological diagnosis.

## Compound mutants

A substantial portion of *CSF3R*-mutated CNL cases (up to 30%) express compound mutations constituting of both membrane-proximal and truncation mutations on the same allele^121^. Because it was unclear whether compound mutants were sufficient to induce leukemogenesis in CNL, Rohrabaugh et al. recently performed an elegant study demonstrating that *CSF3R* compound mutations induced aggressive lethal leukemia in mice and that BaF3 cells expressing *CSF3R* compound mutations in vitro were resistant to both JAK and SRC inhibitors^123^. Furthermore, they proved that both proximal and compound *CSF3R* mutations relied on enhanced MAPK signaling for oncogenic transformation and effectively abrogated leukemia development in mice expressing either of these mutational variants by targeting Mek1/2 with Trametinib^123^. These results not only highlight the potential role of enhanced MAP kinase signaling in certain *CSF3R*-mutated myeloid malignancies but further suggest that patients harboring compound mutations may exhibit resistance to JAK inhibitors in practice^123^.

## Additional mutations in CNL

The majority of patients with *CSF3R*-mutated CNL simultaneously harbor mutations in *SETBP1*, *ASXL1*, or other genes^19,76,126^. Furthermore, ~10–20% of CNL cases are negative for *CSF3R*T618I and other membrane-proximal *CSF3R* mutations suggesting that additional genetic lesions contribute to leukemic phenotype. In parallel, the genomic landscape of CNL is becoming increasingly complex with the recognition of a multitude of other mutations occurring either in conjunction with, or independent of those in CSF3R. In a recent review of the genomics of CNL, Maxson and Tyner comprehensively organized additional genetic lesions in CNL into either mutations in *SETBP1*, signaling mutations such as *JAK2*, spliceosome complex mutations such as *SRSF2* and *U2AF1*, or mutations impacting epigenetics such as *ASXL1* and *TET2*^122^. Mutations in genes such as CALR have also been described in CNL^127^. Below is an outline of the most prevalent mutations in addition to *CSF3R* in CNL and if relevant, their clinical implications.

### SETBP1 mutations

SET is a negative regulator of the tumor suppressor protein phosphatase 2A (PP2A). *Set binding protein 1* (*SETBP1*) protects SET from protease cleavage, thus inhibiting PP2A activity and driving proliferation signals. *SETBP1* overexpression is associated with decreased survival in AML, suggesting a role for *SETBP1* in leukemogenesis^128^. *SETBP1* mutations have been identified in nearly 25% of aCML patients and have been associated with higher white blood cell count at diagnosis and inferior survival;^129^ they have also been identified, to a lesser extent, in MDS/MPN unclassified (10%)^129^, CMML (14.5%), and AML (<1%)^130^. The prevalence of *SETBP1* mutations in CNL has been reported to be between 14% and ~56%^19,76,121,126,131^ and these mutations are enriched in CNL patients harboring *CSF3R* mutations^121^. Meggendorfer et al., Piazza et al., Pardanani et al., Elliott et al., Cui et al., and Gotlib found SETBP1 mutational frequencies of 14%^19^, 25%^129^, 33%^76^, 38%^18^, 42.9%^126^, and 56%^121^, respectively, in their series of CNL patients. With respect to the high prevalence of co-occurring *SETBP1* and *CSF3R* mutations in CNL, Pardanani et al. and Cui et al., respectively, found that 40%^76^ and 75%^126^ of *CSF3R*T618I-mutated CNL patients co-expressed mutations in SET binding protein. Elliott et al. demonstrated that all five (100%) of 13 CNL patients carrying a *SETBP1* mutation co-expressed a *CSF3R*T618I mutation^18^. In a recent review of 16 WHO-defined CNL patients, all of whom harbored the *CSF3R*T618I mutation, *SETBP1* mutations were identified in 63% (10/16)^132^. Conversely, Meggendorfer et al. found that, although not statistically significant, *SETBP1* and *CSF3R* mutations appeared to be mutually exclusive in their cohort of CNL patients (0 of six)^19^.

The prognostic relevance of concurrent *SETBP1* and *CSF3R* mutations remains to be clarified, though some evidence suggests this may be a mutationally detrimental combination. Pardanani et al. revealed a trend toward reduced survival in their cohort of double *CSF3R*-*SETBP1* mutants; however, the analysis was limited by a small number of cases and requires validation^76^. Elliott et al. observed that of the two of 14 CNL patients who developed blast phase disease, both carried *CSF3R*-*SETBP1* (but not *ASXL1*) mutations, suggesting a role for *SETBP1* mutations in blastic transformation^18^. Furthermore, in a report of a patient harboring both *CSF3R*T618I and *SETBP1*D868N mutations, Lasho et al. described resistance to treatment with JAK1 and JAK2 inhibitors in vitro and lack of clinical response to Ruxolitinib in vivo^133^. Similarly, Ammatuna et al. affirmed unresponsiveness to Ruxolitinib in a patient with *CSF3R*-*SETBP1*-mutated disease, though this individual had aCML^134^. In contrast, another case of CNL harboring both *CSF3R* and *SETBP1* mutations treated with Ruxolitinib showed hematological, clinical, and molecular improvement with reductions in both mutational allele burdens—although this patient died approximately 9 months after initiating treatment^135^. Finally, a recent meta-analysis by Shou et al. demonstrated a lack of prognostic impact of *SETBP1* mutations in CNL, although these mutations were significantly associated with poor prognosis in MDS and CMML^136^.

### Cell-signaling genes

#### *JAK2*V617F mutations

There have been several reports of *JAK2*V617F mutations in individuals with suspected CNL bearing typical features of the disease and lacking those of PV, ET, and PMF^76,77,90,137–146^. This putative association, however, must be interpreted in light of the fact that the *JAK2*V617F mutation was often the reason for reporting and that importantly, most of these provisional cases were not tested for oncogenic *CSF3R* mutations as these were not yet described. Using the newly available data on CSF3R mutational status and updated WHO definitions of CNL, *JAK2*V617F and *CSF3R*T618I mutations now appear to be mutually exclusive^76^. A more recent report of 14 cases of strictly WHO-defined, *CSF3R*-positive CNL showed an absence of *JAK2*V617F mutations in all patients tested^18^. Another series by Gotlib et al. showed one patient with concurrent *CSF3R*G683R and *JAK2*V617F mutations but the clonality of this double mutant could not be verified due to limited material^121^.

Nonetheless, rare cases of WHO-defined CNL may harbor JAK2 mutations. In a report of 35 cases of clinically suspected CNL in whom *JAK2*V617F screening was performed, three individuals harbored *JAK2*V617F mutations but only one with truly WHO-defined CNL (but lacking the prototypical *CSF3R* mutation)^76^, supporting the apparent mutual exclusivity of *JAK2* and *CSF3R* mutations, yet allowing for JAK2 positivity in exceptional cases of CNL. Though the *JAK2*V617F mutation serves as a marker of myeloid neoplasm and effectively establishes clonality, its prognostic implications in CNL are unclear. There is some evidence for a favorable impact with reported cases surviving beyond 2 years (and one surviving beyond 8 years treated with Hydroxyurea alone)^141^. However, other data have depicted JAK2-mutated CNL cases with a more aggressive evolution and a high risk of transformation to AML^90,138,143^, confounding the prognostic relevance of this marker. There is thus currently a lack of convincing data to support any diagnostic or prognostic role of *JAK2* mutations in CNL, other than in corroborating clonality.

### Spliceosome-associated genes

#### SRSF2 and U2AF1

Mutations in spliceosome complex components such as SRSF2 and U2AF1 have uncommonly been reported in CNL. Meggendorfer et al. found *SRSF2* mutations to be present in CNL with a mutational frequency of 21%^19^. In a CNL case in which mutational analysis was performed, Senin et al. demonstrated mutations in *CSF3R*T618I, *SETBP1*G870S and *SRSF2*P95H^147^. Further, Dao et al. evaluated 10 cases of *CSF3R*T618I-positive myeloid neoplasms including aCML, CNL, or MPN not otherwise specified and unexpectedly found frequent mutations in *U2AF1* (four of 10 cases of chronic myeloid neoplasms, not defined further in this study)^148^, which had previously been observed in primary myelofibrosis, MDS, and CMML^130^. The cooperative role of these and other spliceosome mutations in the pathogenesis of CNL, as well as their prognostic implications remain to be determined.

### Epigenetic modifiers

#### ASXL1

ASXL1 regulates histone modification and has been shown to be mutated with variable but, in certain cases, high frequency in CNL. Reported mutational frequencies of *ASXL1* mutations in CNL range from 30% to 81%^18,19,132,148^. Pardanani et al. identified *ASXL1* mutations in the majority of *CSF3R*-mutated CNL patients (unpublished)^131^. The most common *ASXL1* mutations observed are frameshift and non-sense mutations in exon 12 resulting in truncation of the gene product. In a critical study by Elliott et al., the presence of *ASXL1* mutations (along with thrombocytopenia) was found to be independently predictive of shortened survival on multivariate analysis^18^. Furthermore, evolution of CNL to CMML was observed in patients harboring *ASXL1* mutations and lacking *SETBP1* suggesting a pathogenic role for *ASXL1* mutations in transformation to CMML^18^.

### TET2 mutations

The TET proteins are dioxygenases that play a role in DNA methylation and somatic mutations in *TET2* are frequently present in myeloid and, to a lesser extent, lymphoid malignancies^149^. In their cohort of 14 CNL patients, Meggendorfer et al. found that close to 30% of them were *TET2*-mutated^19^. Interestingly, concomitant *SRSF2* and *TET2* mutations were not observed in any case, though this was not statistically significant^19^. In a recent report by Dao et al., a CNL patient negative for the *CSF3R* mutation but still meeting strict WHO-defined criteria for CNL harbored a *TET2* mutation along with potentially pathogenic mutations in *PTPN11* (a non-receptor tyrosine kinase), *TP53*, *DNMT3A*, *NF1*, *BCOR*, *BCORL1*, *PHF6*, and *RUNX1*, as revealed by NGS^150^. Sporadic mutations in other epigenetic modifiers such as *EZH2* and *KDM6A* have also been reported in CNL^148^.

In recent years, the involvement of *TET2* and *ASXL1* mutations in clonal hematopoiesis of indeterminate potential (CHIP)^151^ and the current observation that such mutations frequently co-occur with *CSF3R* mutations in CNL (sometimes with mutational frequencies up to 81% as is the case for *ASXL1*) has led to the theory that CHIP may provide a backdrop for a later-occurring *CSF3R* mutation which, in turn, imparts a neutrophilic phenotype resulting in CNL^122^. In their recent review on CNL genomics, Maxson et al. provide three distinct models for mutation acquisition in CNL, each with their own diagnostic, therapeutic and prognostic implications, with *CSF3R* mutations occurring as either a primary or secondary event^122^. This is a fascinating potential association and more data will be required in order to fully elaborate its mechanistic basis.

### Calreticulin

Mutations in calreticulin are present in the majority of *JAK2/MPL*-negative cases of ET and PMF^152,153^. Lasho et al. provided the first account of *CALR* mutations in CNL in 2014 with their observation of a CNL patient harboring *CSF3R*T618I, *SETBP1*G870D, and a novel *CALR* point mutation (*CALR*E398D c.1194 G>T)^127^. This variant was distinct from the classical *CALR* mutations found in ET or PMF, suggesting an alternate pathogenetic mechanism. Another series of CNL patients in the Mayo Clinic cohort also included one patient carrying the same distinct *CALR* mutation (E398D)^18^. Cui et al., in their study of 14 WHO-defined CNL patients found eight expressing the *CSF3R*T618I mutation, including one with a concurrent *CALR* exon 9 frame-shift mutation (c.1154–1155insTTGTC)^126^. Thus far, there are no clear prognostic or therapeutic implications of *CALR* mutations in CNL, although the *CALR* mutant may become an interesting and relevant therapeutic target in the future.

### Additional mutations

By using exome and RNA sequencing, Menezes et al. sought to identify candidate pathogenic genes in CNL and demonstrated an array of genomic lesions^154^. In addition to the classical *CSF3R*T618I mutation, mutations in genes involved in splicing machinery (*LUC7L2* and *U2AF1*), protein kinases (*PIM3*-*SCO2* fusion gene), and epigenetic regulation (*TET2* and *ASXL1*) were identified in an index CNL patient.

The PIM3 oncogene belongs to the Serine-Threonine protein kinase family (PIM subfamily) and plays a role in regulating signal-transduction cascades driving cell proliferation and survival^154^. Mutations in *PIM3* have been found to be enriched in hematological and epithelial tumors^154^. These results raised an interest for “cooperating mutations” and provided a rationale that these additional mutations may serve as new therapeutic targets for agents such as PIM kinase inhibitors. Though *LUC7L2* mutations have previously been described in MDS, they have rarely been identified in CNL and their putative pathogenic impact remains uncertain^155^.

More recently, Langabeer et al. reported the results of NGS studies on a modest but informative cohort of four *CSF3R*T618I-mutated CNL patients, identifying additional mutations in all those tested: *SRSF2* (*n* = 4/4), *SETBP1* (*n* = 3/4), *NRAS* (*n* = 1), and *CBL* (*n* = 1)^156^.

## Clonal evolution

The acquisition of additional molecular or cytogenetic abnormalities during clonal evolution has been reported in CNL^18,156^. The abovementioned study by Langabeer et al. using NGS also included analyses at the time of blastic transformation which identified clonal evolution in all patients as evidenced by increasing *CSF3R*T618I allele frequency or gain/loss of mutations^156^. The finding of *NRAS* mutations both at diagnosis and during blast crisis in this study provides a rationale for use of MEK inhibitors in CNL^156^. In the CNL patient having transformed to blastic phase in a study by Elliott et al., new karyotypic aberrations in the form of acquisition of monosomy 5 and 7 were also noted^18^. Another report additionally noted trisomy 21, deletion 12p, and monosomy 7 at the time of blast transformation in three of 12 CNL patients who were cytogenetically normal at baseline, though treatment with Hydroxyurea was postulated to have contributed to these karyotypic changes^12^. Recently, Nooruddin et al. provided valuable insight into the clonal evolution of CNL as acquisition of de novo mutations in *KIT* and *GATA2* as well as increase in *RUNX1* allele frequency were present in an index CNL patient at the time of disease progression and are posited to have driven clonal evolution^135^.

## Mutation order

The order of acquisition of *CSF3R* mutations relative to additional mutations in *SETBP1*, epigenetic modifiers, or spliceosome machinery has been determined only in isolated case reports and further studies are required in order to understand the impact of mutation chronology on the clonal evolution and progression of CNL.

## Revised 2016 WHO diagnostic criteria

In 2016, the WHO classification of hematologic malignancies was updated to reflect the changes in the genomic landscape of CNL, endorsing the *CSF3R* mutation’s “strong association” with CNL^38^. This justly represented the most significant modification with respect to the 2008 criteria. The presence of *CSF3R*T618I or another activating *CSF3R* mutation now comprises one of the five key diagnostic components of CNL. While these disease-defining mutations serve as diagnostic markers if present, the WHO also allows for the possibility of *CSF3R*-negative disease in the face of chronic neutrophilia, splenomegaly, and the exclusion of reactive leukocytosis^38^. Though the absence of *CSF3R* mutation does not exclude the possibility of CNL, however, it should prompt careful review of the diagnosis and consideration of alternative diagnoses.

The rest of the updated 2016 WHO diagnostic criteria for CNL remain relatively unchanged and are summarized in Table 1. The criteria include: peripheral blood leukocytosis of ≥25 × 10^9^/L (of which >80% are segmented neutrophils plus band forms and <10% are neutrophil precursors with rare myeloblasts), monocyte count <1 × 10^9^/L, and absence of dysgranulopoiesis, as well as hypercellular bone marrow with granulocyte hyperplasia, normal maturation, and <5% myeloblasts^38^. The remaining components are exclusionary and consist of absence of WHO criteria for another MPN and absence of rearrangements in *PDGFRA*, *PDGFRB*, *FGRF1*, or the newly added *PCM1-JAK2*^38^. If there is an underlying plasma cell disorder, myeloid clonality must be demonstrated in order to make the additional diagnosis of CNL.

**Table 1:** World Health Organization 2016 Revised Diagnostic Criteria for chronic neutrophilic leukemia^a^

1. Peripheral blood WBC ≥25 × 10^9^/L
Segmented neutrophils plus band forms ≥80% of WBC
Neutrophil precursors (promyelocytes, myelocytes, and metamyelocytes) <10% of WBC
Myeloblasts rarely observed
Monocyte count <1 × 10^9^/L
No dysgranulopoiesis
2. Hypercellular bone marrow
Neutrophil granulocytes increased in percentage and number
Normal neutrophil maturation
Myeloblasts <5% of nucleated cells
3. Not meeting WHO criteria for *BCR-ABL1*^+^ CML, PV, ET, or PMF
4. No rearrangement of *PDGFRA*, *PDGFRB*, or *FGFR1*, or *PCM1-JAK2*
5. Presence of *CSF3R*T618I or other activating *CSF3R* mutation
or
In the absence of a *CSFR3R* mutation, persistent neutrophilia (at least 3 months), splenomegaly, and no identifiable cause of reactive neutrophilia including absence of a plasma cell neoplasm or, if present, demonstration of clonality of myeloid cells by cytogenetic or molecular studies

*WBC* white blood cells, *CML* chronic myeloid leukemia, *PV* polycythemia vera, *ET* essential thrombocythemia, *PMF* primary myelofibrosis

^a^ adapted from Arber et al. ^38^

## Prognosis and natural history

Though the clinical course of CNL is variable, the overall prognosis is generally reserved with a substantial portion of patients eventually progressing to blast crisis. CNL has been historically described as having a “fatal clinical course”^11^. Overall median survival has been reported between 21 and 30 months in earlier reports, with a 5-year survival of 28% in a 2002 review by Reilly^6,157^. A more recent series of 16 CNL patients, all of whom harbored the *CSF3R*T618I mutation, demonstrated a median overall survival of 24 months^132^ which is similar to the median overall survival reported from 40 CNL cases by Elliott et al. (23.5 months)^89^. Unfortunately recent survival data ominously mirrors that of historical cohorts, showing little to no improvement over time. The transformation rate to acute myeloid leukemia (AML) has been shown to vary between 10% and 21.2%^6,16^ with a median time to AML transformation of 21 months (3–94 months)^89,158^.

The spectrum of fatal complications in CNL includes hemorrhagic diathesis, with fatal intracranial hemorrhage being particularly common in earlier reports^11^, progressive disease, blastic or leukemic transformation, and treatment-related toxicity following chemotherapy induction or transplantation^12,158^.

Distinct disease phases analogous to the accelerated and blastic phases observed in CML have not formally been defined in CNL, though its natural history often does recapitulate that of untreated CML. Disease progression in CNL typically involves resistance to treatment, progressive refractory neutrophilia, increasing red cell and platelet transfusion dependency, worsening organomegaly consistent with disease acceleration, and eventual blast crisis which to date, has been exclusively reported as myeloid. As discussed, such progression may be associated with the acquisition of additional cytogenetic abnormalities^148^.

Further, as in other classic MPN, there appears to be the potential to evolve toward/from other MPNs such as PV^50,51^ and CMML^18^.

## Prognostic markers

There is currently no prognostic scoring system for CNL and due to the rarity of the disease, appropriately validating one would be anticipated to be challenging. Although it is tempting to extrapolate from other myeloid malignancies in assuming that factors such as advanced age, high blast count, extent of splenomegaly, and severity of cytopenias confer a negative prognosis, at this time there are no data to formally support this in CNL. Nevertheless, certain phenotypic and molecular features including leukocyte and platelet count as well as specific mutations and mutational combinations have emerged as potential prognostic markers in CNL.

Cui et al. recently studied 16 WHO-defined CNL patients and found prognostic relevance of an elevated WBC count above 50 × 10^9^/L^132^. In this series, those presenting with WBC above 50 × 10^9^/L had a median overall survival of 11 months compared with 39 months in those with WBC below 50 × 10^9^/L^132^.

In a critical and comprehensive study, Elliott et al. evaluated the prognostic relevance of a number of factors including age, LDH, cytopenias, splenomegaly, bilirubin levels, and genetic variables such as *SETBP1*, *ASXL1* and *CSF3R* mutations in 14 *CSF3R*-mutated CNL cases^18^. In univariate analysis, survival was detrimentally impacted by the presence *ASXL1* mutations, thrombocytopenia, and increased total bilirubin. However, on multivariable analysis, only the presence of *ASXL1* mutations and thrombocytopenia remained independently adverse prognostic factors of decreased survival^18^. Borderline significance was found for both gender and elevated alkaline phosphatase in this study. Of interest, four (29%) patients were alive after a median follow-up of 77.6 months, none of whom harbored *ASXL1* mutations. The authors concluded that cooperative *ASXL1* mutations were frequent (57%) and independently predictive of a shortened overall survival in in CSF3R-mutated CNL patients, consistent with their negative prognostic role in other myeloid malignancies.

Interestingly, though they may have a pathogenic role in disease transformation and have been shown to confer refractoriness to treatment and a trend toward decreased survival^76,133^, *SETBP1* mutations were not an independent predictor of overall survival in the aforementioned study^18^. A recent meta-analysis by Shou et al. also examined the prognostic significance of *SETBP1* mutations in various myeloid malignancies including CNL^136^. Although they found that MDS and CMML patients exhibiting *SETBP1* mutations had significantly worse prognosis than their wild-type counterparts, there was—similar to Elliott et al.’s findings^18^—no significant effect of *SETBP1* mutations on prognosis in CNL, though both these studies were limited by relatively small sample sizes^136^.

Screening for *ASXL1* and *SETBP1* mutations in both *CSF3R*-mutated and unmutated CNL patients could thus be advocated in light of their potential prognostic significance (for *ASXL1*) and their value in ascertaining clonality.

## Management

Effective management translating to a survival benefit in CNL has been hampered by the rarity of the disease, the lack of prospective trials, and the limited therapeutic options, which have themselves, largely been borrowed from the arsenal of therapies utilized in other MPN. In 2002, Bohm et al. expressed that “because of the rarity of the disease, no therapeutic standard has been determined”^11^ and this remains true to this day as there still is no current standard of care in CNL. Aside from hematopoietic stem cell transplant (HSCT), which itself is restricted to a minor portion of CNL patients, none of the commonly used therapeutic agents provide disease-modifying effects and no significant therapeutic advances have been made to improve this.

Both splenic irradiation and splenectomy have been used since 1979 for the palliation of symptomatic splenomegaly in CNL;^7^ and interestingly, in fact, the index case reported by Tuohy was treated with splenectomy^1^. However, due to reports of worsening neutrophilia post-operatively, splenectomy cannot be recommended as part of the treatment approach for CNL^1,10^.

Reports of use of oral chemotherapy in CNL also date back to the late 1970’s and early 1980’s^7,10,159^. Classically, agents such as Busulfan and Hydroxyurea have been used to temporarily control leukocytosis. Although they may help achieve disease stability for a period of time, however, they inevitably lose their efficacy and offer no curative potential.

Hydroxyurea has historically been the most frequently utilized agent with up to 75% of patients showing an initial response as assessed by reduced leukocytosis and/or splenomegaly with median duration of therapy of 12 months (range: 6–87)^12^. Surprisingly, in this study, two patients continued in the chronic stable phase on Hydroxyurea for 18 and 87 months, respectively^12^. In a 2015 report from the Mayo Clinic of 14 cases of CNL, Hydroxyurea was the most frequently used therapy and was ultimately received by all patients in this series^18^. Despite initially controlling hematological parameters, most patients became refractory, eventually displaying increased transfusion dependence. Furthermore, up to ~25% of cases have been reported to be refractory to such therapy from the outset^12^.

Other therapies in the 2015 Mayo series included Interferon, hypomethylating agents, Ruxolitinib, Thalidomide, Cladribine, and Imatinib^18^ and many other accounts of use of these agents have been published, as they constitute the most common therapeutic approach^6,11,12^. Other therapies that have been attempted historically include low-dose Cytarabine, 6-Thioguanine (6-TG), 2-Chlorodeoxyadenosine, and leukapheresis—typically with short-lived responses^12^. These agents thus provide a palliative and usually transient benefit and all fail to induce remission.

Treatment with some of these agents, including Cladribine, Thalidomide, and Ruxolitinib have also been attempted following refractoriness to Hydroxyurea with, again, typically short-lived responses^12,18^.

## Interferon

Interferon-alpha (IFN-a) has a long history of use in CNL and is the only therapeutic agent to offer the potential for durable remissions as published in limited case reports^11,18,140,157^.

Meyer et al. reported two patients with characteristic features of CNL treated with IFN-a^157^. At initiation of therapy, both patients had progressive disease, including one who was refractory to Hydroxyurea treatment. Both patients achieved long-term stabilization of their disease with IFN therapy duration of 16 and 26 months, respectively. The first patient showed slow progression, though still did not require therapy, after discontinuing IFN (follow-up period 90 months), and the second patient was stable receiving ongoing therapy (follow-up period 66 months). In one of the patients, the dose of IFN could be significantly reduced during maintenance without relapse. Moreover, neither presented infectious or hemorrhagic complications under therapy. This study demonstrated that IFN-a was not only safe and effective in CNL (even in pretreated and actively progressing patients), but that it also could induce durable remissions^157^. A review of 14 cases of CNL by Bohm et al. included a patient treated with IFN-a who maintained a clinical remission for the duration of 41 months, corroborating Meyer’s findings^11^. Similarly, Zhang et al. described a *JAK2*V617F-mutated CNL patient treated with IFN-a whose WBC counts were controlled below 10 × 10^9^/L and who remained clinically well during maintenance IFN treatment (though duration of remission was not specified)^140^. Yet another retrospective series included one patient treated with IFN (along with Hydroxyurea co-treatment) who attained prolonged partial response for at least 2 years, after which IFN was discontinued due to disease progression^18^.

These limited studies must evidently be counter-balanced with the fact that not all patients will respond to or tolerate treatment with interferon^160^, though they do provide a rationale for continuing to include this agent in our therapeutic arsenal for CNL.

## Induction chemotherapy

Unfortunately, despite the aggressivity of induction chemotherapy, it has not been reported to induce hematological remission in CNL. There is one account of a patient in blast phase attaining a second chronic phase following AML induction-type “7 + 3” chemotherapy^10^; however, the majority of patients are either refractory or succumb to treatment-related mortality, most notably complications of cytopenias including intracranial hemorrhage and sepsis^11,12,89^.

## Hematopoietic stem cell transplant

Though the aforementioned therapeutic agents may induce temporary disease stability or, in rare instances, durable remissions as may be the case with IFN, none have yielded disease-modifying effects. As most described cases of CNL occur in elderly patients, with only three cases under the age of 40 years being reported as of 1996, accounts of allogeneic HSCT were conceivably rare in early literature. The first report of HSCT in CNL was in 1996 by Hasle et al. describing two young individuals (15 and 25 years old) treated with allogeneic HSCT^10^. Both were in remission 6.5 and 4.5 years post-HSCT, respectively. Other earlier publications by Bohm et al. and Piliotis et al. reported cases of young patients with remissions of up to 73 months after related allogeneic HSCT^11,88^. Likewise, Goto described the case of a young CNL patient transplanted in stable phase, this time using an unrelated HLA matched donor, with remission persisting beyond 3 years^161^. The majority of reported cases of HSCT in CNL thus far have consisted of sibling donors, though at least two reports including that of Goto, relay cases of unrelated donors^138,161^.

Transplant has been described in all phases of disease. Notably, patients having received transplant in blast phase appear to have experienced worse outcomes with regards to regimen-related toxicity and/or early relapse^12,138^. Kako et al. reported a case of a 46-year-old male with *JAK2*V617F-positive CNL who underwent allogeneic HSCT in progressive phase and developed early systemic relapse and central nervous system infiltration^138^. In a review of transplant outcomes in CNL, it was demonstrated that 71% of patients who received HSCT in chronic phase had an ongoing remission lasting over 7 months compared to shorter remission durations for those transplanted in accelerated phase^158^, altogether suggesting that a more favorable outcome may be achieved when transplantation is undertaken during the chronic phase of disease.

The presence of the *CSF3R* mutation may potentially serve as a tool for monitoring patients post-transplant when present at baseline. Lee et al. described patients with both CNL and CMML harboring *CSF3R*T618I mutations having undergone allogeneic HSCT^162^. In both cases, the *CSF3R*T618I mutation was not detected following allogeneic HSCT as the patients remained in remission, suggesting a predictive role of this mutation in post-transplant relapse^162^. Prior studies showing a correlation between post-transplant relapse and the persistence of the *CSF3R*T618I in aCML support this and endorse monitoring of *CSF3R* mutant allele burden as a marker of transplant efficacy^163^.

Given the limited efficacy of available therapeutic agents—and despite the rare accounts of remissions with less toxic regimens—it is currently recommended that eligible patients be directed toward HSCT. Because the principle of “risk-adapted therapy” does not formally apply to CNL as it does to other MPN, the precept of “transplant if you can” is probably a safe approach in managing this aggressive and often rapidly fatal disease. Though optimal timing of transplant is uncertain, there is some data to preferentially support transplant in chronic phase^12,138,158^. Consequently, it may be advisable to have eligible patients evaluated for transplant early in their disease course, prior to blast transformation. The scarcity of data on outcomes following transplant, particularly when using alternative stem cell sources, such as cord blood, or non-myeloablative approaches constitutes one of the many challenges in CNL and will require a collective effort in order to achieve progress on this front.

## JAK inhibitors

The JAK1/2 inhibitor Ruxolitinib is currently approved for treatment of patients with intermediate- or high-risk myelofibrosis and, more recently, for PV patients intolerant or refractory to Hydroxyurea^164,165^. Due to the pivotal role of the JAK-STAT pathway in oncogenic signaling in CNL, it was reasoned that Ruxolitinib may provide clinical efficacy in this disease. Below we provide a summary of studies evaluating Ruxolitinib in both murine models and humans.

The original study by Maxson et al. included a patient harboring the *CSF3R*T618I mutation who was treated with Ruxolitinib 10 mg orally twice daily^14^. The patients’ cells exhibited sensitivity to Ruxolitinib in vitro and in vivo and clinical effects included a significant reduction in white blood cell and absolute neutrophil count as well as a normalization of platelet count. Further blood count decreases occurred with escalation of the dose to 15 mg orally twice daily. What was initially reported as a 5-month response translated into a longer-term one as the patient was shown to still be responding after 11 months of therapy^121^. The “proof of concept” study by Fleishman et al. also showed a salutary effect of Ruxolitinib in a mouse model transplanted with *CSF3R*T618I-expressing hematopoietic cells^120^. Use of Ruxolitinib in this model suppressed *CSF3R*T618I-induced cell proliferation, controlled leukocytosis, reduced spleen weight, and increased mouse body weight, mirroring the symptomatic improvements seen in humans^120^. In contrast, Elliott et al. reported on a CNL patient with a more mitigated response to Ruxolitinib as the individual eventually required addition of Hydroxyurea in order to achieve disease control^18^.

Three studies evaluated the role of Ruxolitinib in the treatment of CNL patients co-expressing CSF3R and *SETBP1* mutations with varying results^133,135,166^. Lasho et al. described a case of WHO-defined CNL with concurrent *CSF3R*T618I and *SETBP1* mutations failing treatment with Hydroxyurea subsequently treated with Ruxolitinib 10 mg orally daily in addition to Hydroxyurea^133^. The patient became refractory to treatment with both agents, displaying worsening leukocytosis despite Ruxolitinib 20 mg orally twice daily and Hydroxyurea 1 g orally daily. It is notable that Ammatuna described the same refractoriness to treatment in a *CSF3R*T618I and *SETBP1* double mutant, but in WHO-defined aCML^134^. This discrepant lack of efficacy compared to the above-mentioned studies raised the possibility that co-expression of *CSF3R*T618I and *SETBP1* may hamper JAK inhibitor therapy. Counter to this concept however, both Nooruddin et al. and Stahl et al. reported cases of CNL with concurrent *CSF3R*T618I and *SETBP1*G870S mutations initiated on Ruxolitinib titrated up to 20 mg orally daily with periods of response lasting from 5 to 9 months^135,166^. In the former study, the patient noted symptom improvement after only 2 weeks of treatment and in the latter case, the additional presence of the *ASXL1* mutation, known to confer a worse prognosis in CNL, may have biased the study^166^.

Similarly, it was found that harboring compound *CSF3R* mutations, as opposed to isolated proximal or truncation mutations, conferred resistance to Ruxolitinib in a murine model of CNL, suggesting that JAK inhibitors may be of minimal benefit to the ~1/3 of CNL patients exhibiting such compound *CSF3R* mutations^123^.

The question of whether JAK inhibitor therapy could achieve reduction in *CSF3R* allele burden was initially addressed by Dao et al. in an aCML patient harboring a *CSF3R*T618I (along with *CBL*I383T and KDM6AS114C) mutation^167^. Treatment with Ruxolitinib resulted in hematological and symptomatic improvement, although no impact on allele burden was observed, suggesting the unlikeliness of Ruxolitinib having a selective anti-clonal or disease-modifying effect^167^. More recently, however, data have emerged demonstrating reduction in *CSF3R* allele burden consequent to Ruxolitinib therapy. Nooruddin et al. observed a parallel decrease in *CSF3R* and *SETBP1* mutation allele burden in response to Ruxolitinib therapy^135^. In this case, the acquisition of de novo mutations in *KIT* and *GATA2* as well as increase in *RUNX1* allele frequency are postulated to have played a role in disease evolution and/or Ruxolitinib resistance, but this requires validation^135^. Furthermore, Gunawan et al. reported on a patient with CNL harboring compound *CSF3R* mutations (*CSF3R*T618I and *CSF3R*G739 truncation mutation) in whom treatment with Ruxolitinib induced hematological response in parallel with reductions in allelic burden for both mutations^168^. Molecular relapse coincided with hematological relapse at day 784^168^. It is noteworthy that despite initial decline in *CSF3R* allele burden, this patient did not show symptomatic improvement, raising the question of whether reduction in *CSF3R* allele burden translates to a clinically meaningful benefit.

The host of variables, molecular and otherwise, underlying differential responses and eventual refractoriness to Ruxolitinib remains to be formally elucidated. It has been speculated that mechanisms involved in Ruxolitinib resistance in CNL may model those already described by Koppikar et al., namely heterodimeric reactivation of JAK-STAT by other JAK kinases^169^. It is also plausible that an individual’s response (or lack thereof) to treatment with JAK inhibitor therapy is in part molecularly-driven and it will be important to determine how additional mutations such as those in *ASXL1*, *SETBP1*, or *SRSF2* modulate the clinical effect of Ruxolitinib. Correspondingly, the attenuated sensitivity of compound *CSF3R* mutations to Ruxolitinib in certain studies^123^ suggests that additional aberrant signaling pathways are involved, possibly via activation of MAPK downstream signaling by alternative cellular signaling networks.

A prospective multicenter phase II clinical trial evaluating safety and efficacy of Ruxolitinib in CNL patients is currently underway (NCT02092324) and is expected to clarify Ruxolitinib’s role in the management algorithm of CNL. At the present time, considering the dearth of therapeutic options and some encouraging in vivo data, it would be reasonable to consider Ruxolitinib in the therapeutic arsenal for HSCT-ineligible CNL patients^131^.

## Novel therapeutic targets

Apart from the aforementioned study involving Ruxolitinib, there are, at this time, no other investigational agents undergoing study for use in CNL. The finding of *NRAS* mutations in a portion of CNL patients has led to the rationale that treatment with MEK inhibitors such as Trametinib may offer disease control in certain mutationally-defined subsets, as it has been shown to do in other myeloid malignancies including aCML^170,171^. Rohrabaugh et al. supported this concept, showing that in vivo inhibition of MEK1/2 by Trametinib in a murine CNL model expressing compound *CSF3R* mutations effectively abrogated leukemia development^123^. These studies seemingly justify the evaluation of MEK1/2 inhibitors in patients with similarly molecularly annotated CNL. As we gain more insight into the genomics and pathobiology of CNL, it is likely that molecularly driven targeted therapy will increasingly emerge as a compelling treatment approach.

## Conclusion

CNL remains a rare but often clinically aggressive MPN with few effective treatment options. Substantial progress has been made since the first description of the disease, most notably with the discovery of oncogenic *CSF3R* driver mutations in the majority of CNL patients. There now exists a more intricate molecular framework underpinning CNL biology than was originally thought. With the recent findings of additional genetic lesions in a substantial proportion of CNL patients, and considering the lack of disease-defining *CSF3R* mutations in others, one of the critical challenges will be molecularly anatomizing this disease: determining additional driver and ancillary mutations, their cooperativity, their interactions with cytogenetic and other extrinsic factors, and their phenotypic, therapeutic, and prognostic implications—and ultimately integrating these variables into an operationally useful schema for prognostication and treatment. The order of mutation acquisition in CNL will also be important to define as it too may impact prognosis and response to therapy.

The new WHO diagnostic criteria reflect the strides in our knowledge of CNL pathogenesis as well as our increasingly sophisticated and available molecular diagnostic tools. Molecularly annotated sub-categories of CNL are emerging, contingent upon whether patients are *CSF3R*-negative, *CSF3R*-positive (proximal or truncation), compound-mutated, or carriers of *CSF3R* plus additional genetic lesions, potentially each with their own phenotypic and prognostic relevance. Molecular testing and diagnostic criteria will inevitably evolve in parallel to become more profoundly individualized, justly reflecting the genomic complexity of the disease.

As the collective cohort of CNL patients grows, along with our experience, we hope to achieve an even deeper understanding of the molecular, cytogenetic, and extrinsic factors driving disease initiation, transformation, and response to treatment. The optimal timing of transplant remains to be defined, just as the role of Ruxolitinib needs to be circumscribed in the therapeutic algorithm for CNL. Furthermore, new potentially relevant molecular targets have been identified and should be explored in order to maximize therapeutic alternatives. Finally, it is imperative that these advances in knowledge—this *new* science—not only reshape our vision of CNL but hopefully translate to meaningful and practical improvements in patient management and outcomes.
